# Cigarette Smoke Decreases Airway Epithelial FABP5 Expression and Promotes *Pseudomonas aeruginosa* Infection

**DOI:** 10.1371/journal.pone.0051784

**Published:** 2013-01-22

**Authors:** Fabienne Gally, Hong Wei Chu, Russell P. Bowler

**Affiliations:** Pulmonary Division, Department of Medicine, National Jewish Health, Denver, Colorado, United States of America; Lovelace Respiratory Research Institute, United States of America

## Abstract

Cigarette smoking is the primary cause of Chronic Obstructive Pulmonary Disease (COPD), which is characterized by chronic inflammation of the airways and destruction of lung parenchyma. Repeated and sustained bacterial infections are clearly linked to disease pathogenesis (e.g., exacerbations) and a huge burden on health care costs. The airway epithelium constitutes the first line of host defense against infection and our previous study indicated that Fatty Acid Binding Protein 5 (FABP5) is down regulated in airway epithelial cells of smokers with COPD as compared to smokers without COPD. We hypothesized that cigarette smoke (CS) exposure down regulates FABP5, thus, contributing to a more sustained inflammation in response to bacterial infection. In this report, we show that FABP5 is increased following bacterial infection but decreased following CS exposure of primary normal human bronchial epithelial (NHBE) cells. The goal of this study was to address FABP5 function by knocking down or overexpressing FABP5 in primary NHBE cells exposed to CS. Our data indicate that FABP5 down regulation results in increased *P. aeruginosa* bacterial load and inflammatory cytokine levels (e.g., IL-8) and decreased expression of the anti-bacterial peptide, β defensin-2. On the contrary, FABP5 overexpression exerts a protective function in airway epithelial cells against *P. aeruginosa* infection by limiting the production of IL-8 and increasing the expression of β defensin-2. Our study indicates that FABP5 exerts immunomodulatory functions in the airway epithelium against CS exposure and subsequent bacterial infection through its modulation of the nuclear receptor peroxisome proliferator-activated receptor (PPAR)-γ activity. These findings support the development of FABP5/PPAR-γ-targeted therapeutic approach to prevent airway inflammation by restoring antimicrobial immunity during COPD exacerbations.

## Introduction

Chronic obstructive pulmonary disease (COPD) is a complex disease characterized by an abnormal lung inflammatory response to cigarette smoke (CS) that leads to tissue destruction and airflow obstruction [1]. Acute exacerbations, mainly caused by bacterial and viral infections, are a major cause of hospitalization and death. The health care costs are a serious economic burden [2] and negatively impact patient's quality of life. COPD patients have been shown to be more susceptible to infections than “healthy” smokers [3], [4], [5]. Smoking appears to dampen innate immune responses, and as a consequence, pathogens proliferate and persist in the airways of COPD patients [6]. *Pseudomonas aeruginosa* (*P. aeruginosa*) represents 5–10% of the pathogens that colonize COPD lungs [7], [8] and is not only involved in acute exacerbations, but also contributes to the chronic process of the disease [9]. In addition, several studies have shown that *P. aeruginosa* is the cause of more infections as severity of COPD increases [10], [11], [12].

Located at the interface between the host and the environment, the airway epithelium represents the first line of host defense against pathogens or irritants (e.g., CS). The protective role of the epithelium includes recognition of potentially dangerous particulates and microbes [13], as well as production of various mediators such as proinflammatory cytokines (e.g., interleukin-8, IL-8), mucins, and antimicrobial substances (e.g., β defensin-2) [14]. Production of these inflammatory cytokines and antimicrobial substances is tightly regulated. However, persistent and repeated infections in COPD patients suggest an abnormal epithelial cell function [15], [16].

In previous work using *Caenorhabditis elegans*, we identified the lipid binding protein 7 (lbp-7) to be down regulated after CS exposure and to play a role in innate immunity [17]. The human orthologue of this protein, the fatty acid binding proteins 5 (FABP5), is highly expressed in primary normal human bronchial epithelial (NHBE) cells but is down regulated in COPD patients [17]. Fatty acid binding proteins (FABPs) are a family of small, highly conserved, cytoplasmic proteins that bind long chain fatty acids and other hydrophobic ligands. FABPs are thought to be involved in fatty acid uptake, transport and metabolism [18]. The *FABP5* gene encodes the epidermal fatty acid binding protein and has been found to be upregulated in psoriasis tissue [19]. We knocked down or overexpressed FABP5 in primary NHBE cells to determine FABP5 host defense mechanisms during bacterial infection in the context of CS exposure.

## Materials and Methods

### FABP5 immunohistochemistry

Immunohistochemistry was used to localize FABP5 protein in lung tissues of 5 normal donors and 5 biopsies of COPD donors. Paraffin-embedded lung tissue sections of 5 µm thickness were deparaffinized in xylene and rehydrated through graded ethanol. Citrate buffer (pH 6.0) was used for antigen retrieval at sub-boiling temperature. Sections were then treated with 0.3% hydrogen peroxide in 0.05M Tris buffered saline (TBS pH 7.6) for 30 min to inhibit endogenous peroxidase. After blocking with 1% normal rabbit serum (Vector Laboratories, Burlingame, CA), the slides were incubated with rat anti-human FABP5 primary antibody MAB3077 (R&D Systems, Minneapolis, MN) or a rat IgG control overnight at 4°C, followed by incubation with biotinylated secondary antibody. Avidin-biotin-peroxidase complex (Vector Lab, Burlingame, CA) was added to the slides for 30 min at room temperature. Thereafter, 0.03% aminoethylcarbazole (AEC) solution with hydrogen peroxide was used for chromogen reaction.

### *P. aeruginosa* culture

*P. aeruginosa* strain used was PAO1-GFP (green fluorescent protein) (resistant to chloramphenicol, 50 µg/ml; kindly provided by M. Schurr, University of Colorado, Denver). PAO1-GFP strain was stored as a stock at −80°C. For each set of experiments, bacteria were streaked onto a Luria-Bertani (LB) agar medium plate and cultured for 18 to 22 h at 37°C. An individual colony was cultured in LB medium and then amplified in a larger volume to prepare aerated, log-phase bacteria by rotary shaking at 37°C until 1×10^8^ CFU/ml was achieved as determined by spectrophotometry (optical density at 600 nm = 0.5). CFU of bacteria were quantified by plating serial dilutions on LB agar medium.

### Primary normal human bronchial epithelial (NHBE) cell culture

Deidentified human lungs not suitable for transplantation were donated to medical research through the International Institute for the Advancement of Medicine (Edison, NJ). All donors or next of kin gave written informed consent for their organs to be used for medical research. We know the age, gender, race, smoking history, cause of death, very brief medical history, and medications at the time of death. Primary normal human bronchial epithelial cells were isolated from bronchial tissues of three donors without any lung diseases or smoking history.

The cells were transduced with either pLL3.7-shFABP5 or pLL3.7-shFirefly luciferase and were cultured under air liquid interface (ALI) conditions to determine if cigarette smoke (CS) and *P. aeruginosa* infection outcomes are affected by gene knockdown of FABP5. Additionally, NHBE cells were transduced with either pLL3.7-FABP5 or pLL3.7-GFP to determine whether FABP5 overexpression protects epithelial cells from *P. aeruginosa* infection. Successful knockdown and overexpression of FABP5 in primary NHBE cells are illustrated in Figure S1. ALI cultures were performed by plating the lentivirus-transduced epithelial cells onto collagen-coated 12-well transwell plates at 4×10^4^ cells/cm^2^, as previously reported [20]. Non transduced cells were used to determine the effects of PPAR-γ inhibition on IL-8 and β defensin-2 production. On day 13 of ALI culture, GW9662 (10 µM), a well characterized PPAR-γ antagonist, was applied on both the apical and basolateral sides of the cells. 0.01% of DMSO was applied on control cells. On day 14 of ALI culture, cells were exposed to CS or air and immediately treated with *P. aeruginosa* (10 cfu/cell) or cell culture medium (control). Two hours post treatment, cells and apical supernatants were collected to measure levels of intracellular bacteria, IL-8, β defensin-2 and PPAR-γ activity. A preliminary study determined that 2 hours of *P. aeruginosa* infection was the best compromise to observe an effect on IL-8 and β defensin-2 levels without increasing bacteria-induced cell death.

### Whole cigarette smoke (CS) exposure system of primary NHBE cells

Whole CS was generated by the consumption of one tobacco cigarette (research reference cigarettes 2R4F, University of Kentucky) using a peristaltic pump and diluted in 1 liter of air. Primary NHBE cells from normal donors were exposed to filtered air or whole CS using a chamber described previously [21]. Nicotine levels in the apical supernatant of cells were measured at 12.5±0.4 µg/ml using a GM/MS method, which are similar to the nicotine levels found in human smokers [22].

### Inoculation of NHBE cells with *P. aeruginosa*

One day prior to bacterial inoculation, the medium of NHBE cells was replaced with infection medium without antibiotics. Bacteria were resuspended in the infection medium at 4×10^5^ cfu/50 µl and were applied to the apical compartment of the Transwell membrane insert.

### Recovery of intracellular and extracellular adherent bacteria

After 2 hours of incubation with bacteria, NHBE cells on supported membranes were washed twice with sterile PBS. The membranes were then cut out of the plastic support and transferred to a 1.5 ml eppendorf tube containing lysis buffer (0.25% Triton X-100 in PBS with anti-proteases). Cell lysates were diluted and plated on LB agar plates and incubated (18 h, 37°C) for subsequent determination of intracellular and adherent extracellular colony forming units (cfu).

### IL-8 ELISA

IL-8 was measured using the human IL-8 Duo-Set Immunoassay (R&D Systems, Minneapolis, MN, US) following manufacturer's instructions.

### Real-time reverse transcriptase-PCR

Real-time reverse transcriptase PCR was performed as previously described [23]. Briefly, total RNA was extracted using TRIzol reagent (Invitrogen, Carlsbad, CA) and treated with DNase I (Ambion, Austin, TX). Reverse transcription was performed using 1 µg of total RNA and random hexamers in a 50 µl reaction according to the manufacturer's instructions (Applied Biosystems, Foster City, CA). 18S rRNA was used as the housekeeping gene. The threshold cycle was recorded for each sample to reflect the mRNA expression levels. The comparative threshold cycle method was used to demonstrate the relative expression level of the gene of interest.

### Peroxisome proliferator activated receptor (PPAR)-γ activity assay

Cells were lysed to extract nuclear proteins following manufacturer's instructions (Active Motif, Carlsbad, CA). Nuclear proteins (5 µg per sample) were used to perform the PPAR-γ ELISA (Active Motif), which measures PPAR-γ activation levels. Briefly, multiple copies of PPAR-γ specific double-stranded oligonucleotide were immobilized to a 96-stripwell plate. Nuclear proteins were then added to the well where activated PPAR-γ bound specifically to the oligonucleotide at its consensus binding site. Thereafter, a primary antibody specific for the activated form of PPAR-γ was added, followed by incubation with a HRP-conjugated secondary antibody. A developing solution was then added to provide an easily quantified, sensitive colorimetric readout (e.g., optical density).

### Statistical analysis

Normally distributed data are presented as means ± SEM. One-way analysis of variance (ANOVA) was used for multiple comparisons, and a Tukey's post hoc test was applied where appropriate. Student's *t* test was used when only two groups were compared. Non-parametric (non normally distributed) data are expressed as means and compared using the Mann Whitney test between the two groups. A *p* value <0.05 was considered statistically significant.

## Results

### FABP5 is down regulated in airway epithelial cells of COPD patients

**Figure 1:**
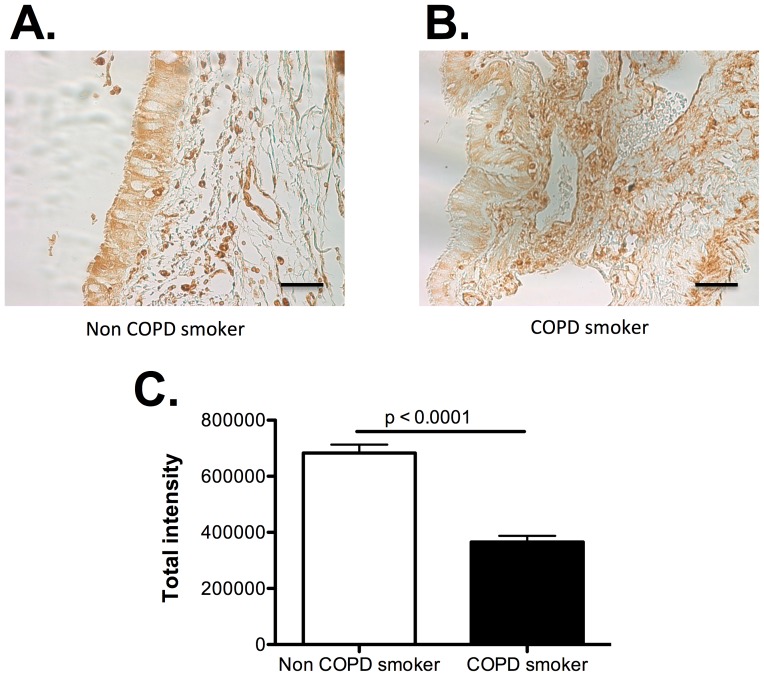
FABP5 expression is decreased in COPD airway epithelial cells. **A.** Immunostaining of FABP5 protein (brown color) in airway epithelial cells from normal smoker. Bar represent 50 µm. **B.** Immunostaining of FABP5 protein (brown color) in airway epithelial cells from COPD smoker. Bar represent 50 µm. **C.** FABP5 protein was quantified by densitometry using the Scion Image software (Scion Corporation, Frederick, MD). Data are representative of 5 different subjects per group.

Figure 1 shows that FABP5 immunostaining of human lung tissue is strongly positive in small airway epithelial cells of the normal lung (Figure 1A), but reduced in COPD airway epithelial cells (Figure 1B). These data confirm our previous study where we have shown that human brushed bronchial epithelial cells express FABP5, and that FABP5 mRNA levels were significantly lower in smokers with COPD than smokers without COPD [17].

### Cigarette smoke (CS) exposure inhibits bacteria-induced FABP5 expression

**Figure 2:**
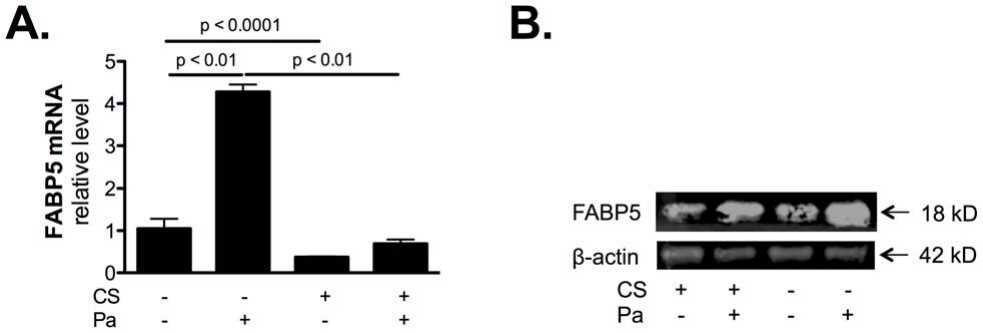
CS exposure prevents bacteria-induced FABP5 expression. **A.** FABP5 mRNA expression is increased post *P. aeruginosa* infection, however CS exposure decreases FABP5 mRNA expression and prevents bacteria-induced FABP5 mRNA expression. **B.** Representative Western blot detection of FABP5 and β-actin proteins shows that FABP5 protein levels increased post Pa infection but CS exposure decreases Pa-induced FABP5 protein levels. Data are representative of 3 independent experiments and are expressed as mean ± SEM.

As indicated in Figure 2, *P. aeruginosa* infection increases FABP5 mRNA (Figure 2A) and protein (Figure 2B) expression. However, CS exposure decreased FABP5 expression and prevented bacteria-induced FABP5 mRNA (Figure 2A) and protein (Figure 2B) expression. This result indicates that FABP5 expression is positively regulated during bacterial infection, but CS exposure prevents FABP5 up regulation.

### CS exposure increases bacterial load and inflammatory cytokine production

**Figure 3:**
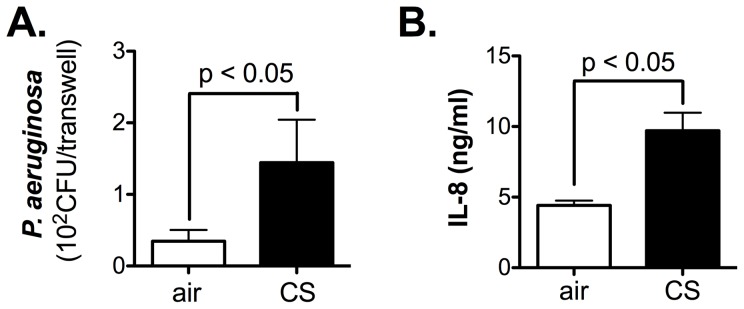
CS exposure impairs *P. aeruginosa* clearance. **A.** CS exposure increases *P. aeruginosa* colony forming units (CFU) on primary NHBE cells. **B.** CS exposure significantly enhances IL-8 secretion in the basolateral supernatant of primary NHBE cell cultures. Data are representative of 3 independent experiments and are expressed as mean ± SEM.

CS exposure increased bacterial load (Figure 3A) as well as IL-8 secretion from primary NHBE cells (Figure 3B). This observation confirms that CS exposure dampens airway epithelial cell host innate immunity.

### FABP5 down regulation inhibits NHBE cells innate immunity

**Figure 4:**
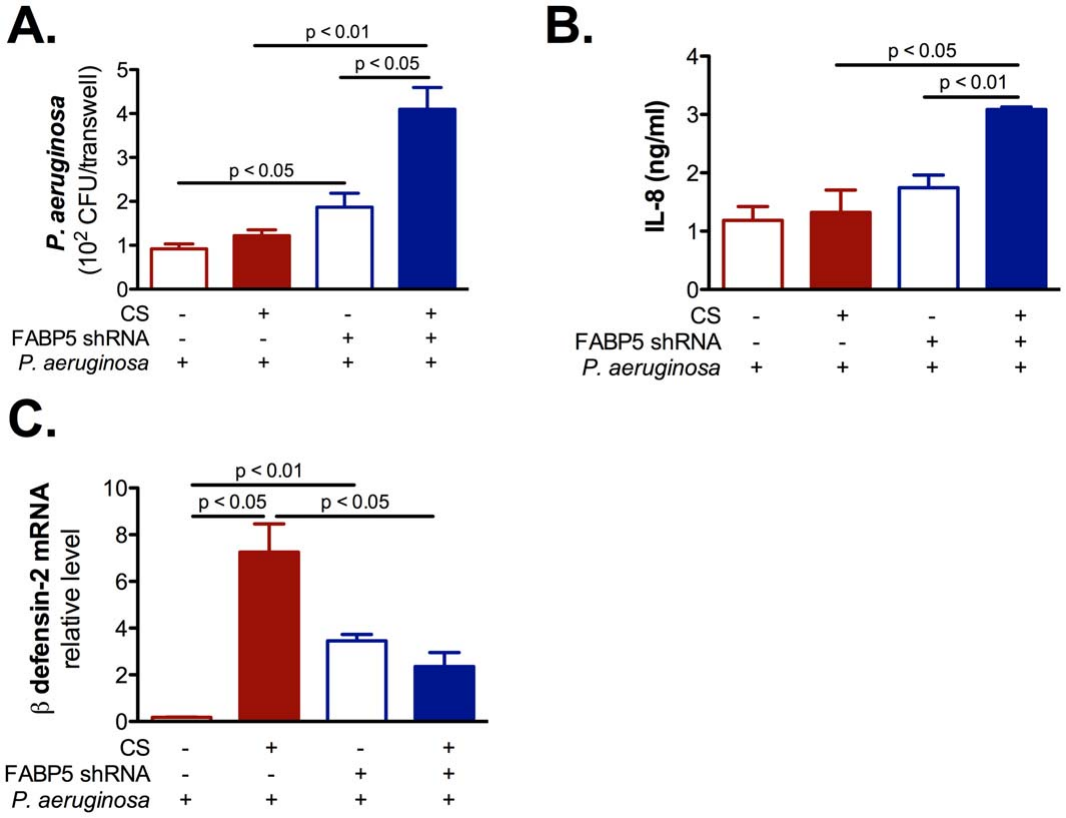
FABP5 down regulation increases inflammation but decreases innate immunity in primary NHBE cells. **A.** CS exposure increases *P. aeruginosa* colony forming units (CFU) on FABP5 knocked down primary NHBE cells. **B.** CS exposure significantly enhances IL-8 secretion in the basolateral supernatant of primary NHBE cell cultures knocked down for FABP5. **C.** CS exposure decreases β-defensin 2 mRNA expression on FABP5 knocked down primary NHBE cells. FABP5 shRNA − indicates cells that were knocked down for Firefly luciferase and FABP5 shRNA + indicates cells that were knocked down for FABP5. Data are representative of 3 independent experiments and are expressed as mean ± SEM.

To understand the role of FABP5 in CS-exposed primary NHBE cells innate immune responses, we knocked down FABP5, exposed the cells to CS and immediately after infected them with *P. aeruginosa*. We found that cells knocked down for FABP5 had higher levels of *P. aeruginosa* CFUs (Figure 4A), higher levels of IL-8 (Figure 4B), but lower levels of β defensin-2 mRNA (Figure 4C) than cells knocked down for Firefly luciferase. These results suggest that FABP5 knock down sensitizes CS-exposed cells to *P. aeruginosa* infection, which provides a direct link between FABP5 and NHBE cell innate immunity.

### FABP5 up regulation increases NHBE cells innate immunity

**Figure 5:**
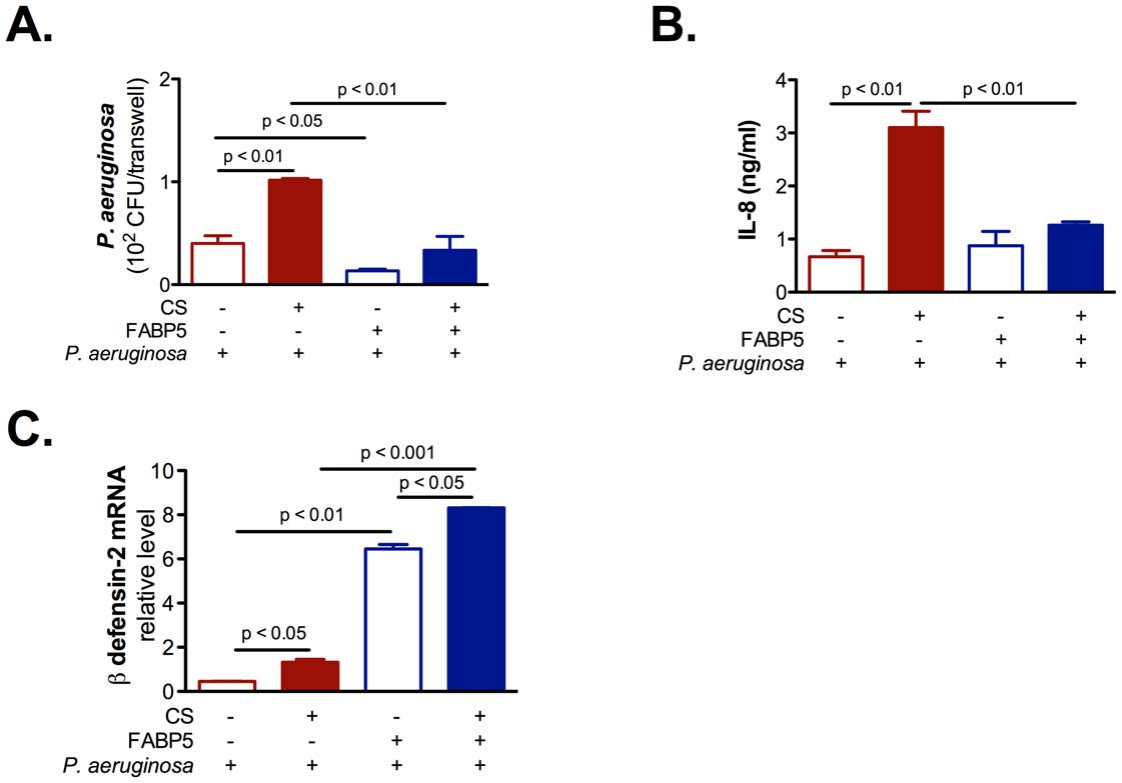
FABP5 overexpression decreases inflammation and increases innate immunity in primary NHBE cells. **A.** CS exposure decreases *P. aeruginosa* colony forming units (CFU) on primary NHBE cells overexpressing FABP5. **B.** FABP5 overexpression blocks CS-induced IL-8 secretion in the basolateral supernatant of primary NHBE cell cultures. **C.** FABP5 overexpression augments β-defensin 2 mRNA expression in primary NHBE cells. FABP5 − indicates cells overexpressing GFP and FABP5 + indicates cells overexpressing FABP5. Data are representative of 3 independent experiments and are expressed as mean ± SEM.

To complement the study described above, we overexpressed FABP5 in NHBE cells. We exposed the cells to CS and infected them with *P. aeruginosa*. We found that cells overexpressing FABP5 had lower levels of *P. aeruginosa* CFUs (Figure 5A), lower levels of IL-8 (Figure 5B), and higher levels of β defensin-2 mRNA (Figure 5C) than cells overexpressing the empty vector. Taken together, these results confirm the protective role of FABP5 during bacterial infection in the context of CS exposure.

### FABP5 expression modulates Toll-like receptors (TLR) 2 and 4 expression

**Figure 6:**
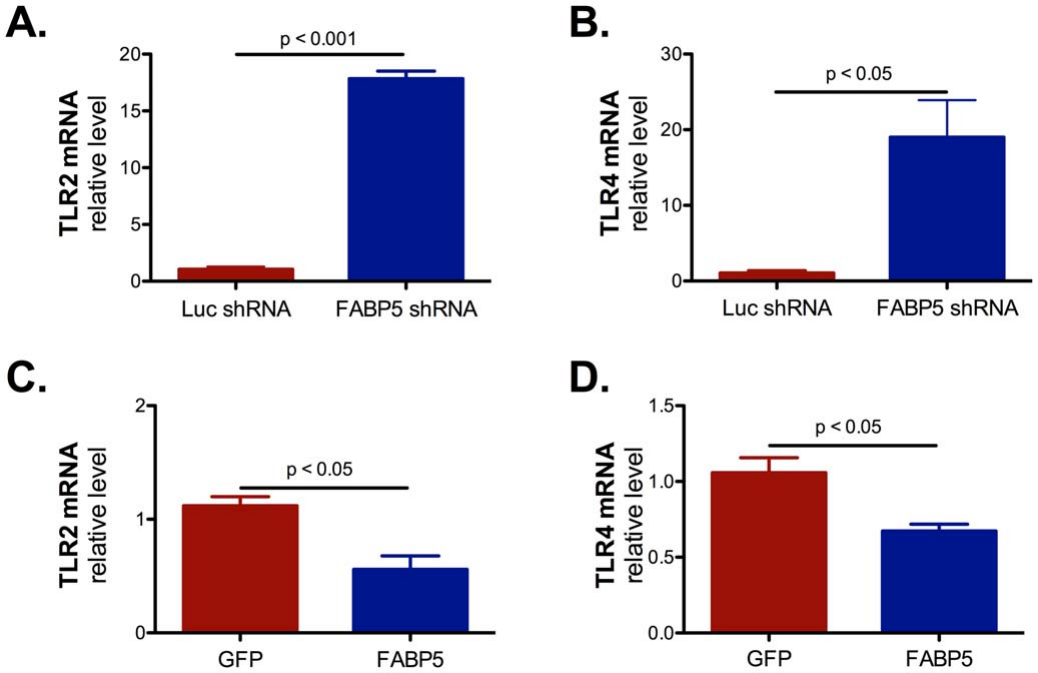
FABP5 expression modulates TLR2 and TLR4 mRNA expression. **A.** TLR2 mRNA expression was induced in cells knocked down for FABP5. **B.** TLR4 mRNA expression was induced in cells knocked down for FABP5. **C.** TLR2 mRNA expression was decreased in cells overexpressing FABP5. **D.** TLR2 mRNA expression was decreased in cells overexpressing FABP5. Data are representative of 3 independent experiments and are expressed as mean ± SEM.

To understand how FABP5 modulated inflammatory cytokine production and β defensin-2 expression, we first measured TLR2 and TLR4 mRNA expression. Indeed, TLR2 and TLR4 have both been implicated in IL-8 and β defensin-2 expression [24], [25]. We determined that down regulation of FABP5 greatly increased TLR2 and TLR4 mRNA expression (Figure 6A and 6B). On the contrary FABP5 overexpression decreased TLR2 and TLR4 mRNA expression (Figure 6C and 6D). These results are in accordance with increased IL-8 secretion when FABP5 is down regulated and decreased IL-8 secretion when FABP5 is overexpressed, but are in contradiction with our observations concerning β defensin-2 expression.

### FABP5 expression modulates peroxisome proliferator activated receptor (PPAR)-γ activity

**Figure 7:**
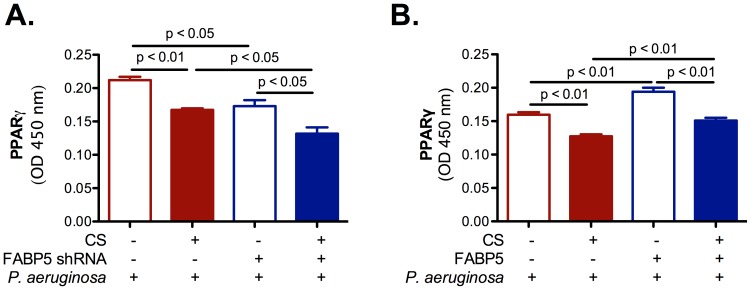
FABP5 expression modulates PPAR-γ activity. **A.** FABP5 down regulation significantly decreases PPAR-γ activity in primary NHBE cell cultures. **B.** FABP5 up regulation significantly increases PPAR-γ activity in primary NHBE cell cultures. FABP5 shRNA − indicates cells that were knocked down for Firefly luciferase. FABP5 shRNA + indicates cells that were knocked down for FABP5. FABP5 − indicates cells overexpressing GFP. FABP5 + indicates cells overexpressing FABP5. Data are representative of 3 independent experiments and are expressed as mean ± SEM.

We then assessed PPAR-γ activity in primary NHBE cells. PPAR-γ is a member of the nuclear hormone receptor superfamily of ligand-activated transcription factors. FABP5 has recently emerged as an important regulator of fatty acid metabolism and as a regulator of PPAR-γ activity. PPAR-γ agonists have been shown to inhibit inflammatory cytokine production and to increase innate antimicrobial immunity in the colon [26]. Thus we hypothesized that CS-induced FABP5 down regulation in airway epithelial cells increases inflammation and infection susceptibility by decreasing PPAR-γ activity. Here we show that CS exposure decreases PPAR-γ activity regardless of the treatment. PPAR-γ activity is decreased in cells knocked down for FABP5 (Figure 7A), but increased in cells that overexpress FABP5 (Figure 7B). Thus, it appears that PPAR-γ activation is required for maintenance of innate antimicrobial immunity and decreased inflammation in the airway epithelium.

### PPAR-γ inhibition decreases NHBE cells innate immunity

**Figure 8:**
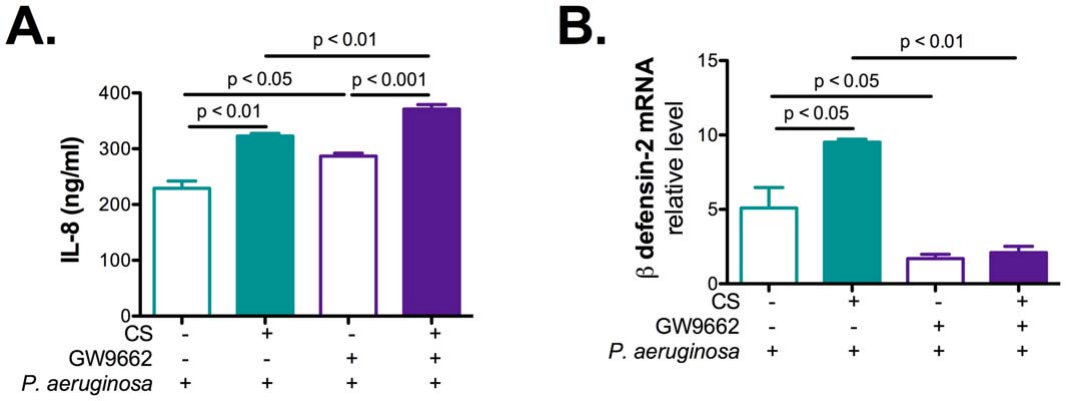
PPAR-γ inhibition increases IL-8 secretion and decreases β-defensin 2 expression in primary NHBE cells. **A.** PPAR-γ inhibition by GW9662 increases IL-8 secretion in the basolateral supernatant of primary NHBE cell cultures. **B.** FABP5 PPAR-γ inhibition by GW9662 dcreases β-defensin 2 mRNA expression in primary NHBE cells. Data are representative of 3 independent experiments and are expressed as mean ± SEM.

To confirm the role of PPAR-γ in FABP5 mediated effect on IL-8 and β defensin-2 secretion, a complementary approach of inhibiting PPAR-γ using GW9662 antagonist was used. Results presented in Figure 8 indicate that inhibition of PPAR-γ resulted in increased levels of IL-8 secretion upon CS and *P. aeruginosa* infection (Figure 8A) and reduced levels of β defensin-2 mRNA (Figure 8B) as compared to cells treated with DMSO alone.

## Discussion

We have previously shown that FABP5 is highly expressed in airway epithelial cells, but is reduced in smokers with COPD as compared to smokers without COPD [17]. However, FABP5 function in airway epithelial cells is still unknown. To elucidate the role of FABP5 in airway epithelial cell host defense, we knocked down or overexpressed FABP5 in primary normal human bronchial epithelial (NHBE) cells. This study confirmed our hypothesis that CS decreases FABP5 expression in airway epithelial cells and contributes to a more sustained bacterial infection. Ablation of FABP5 expression sensitized NHBE cells to CS-induced inhibition of innate immunity whereas FABP5 overexpression protected the cells from CS-induced bacterial infection worsening.

We previously showed that FABP5 is up-regulated in airway epithelial culture upon *Mycoplasma pneumonia* infection, demonstrating its potential role in the innate immune response [17]. CS has been shown to alter a wide range of immunological functions, including innate and adaptive immune responses [27], [28]. In this study, we confirmed that CS inhibits bacteria-induced FABP5 expression, using *P. aeruginosa*, which resulted in increased airway epithelial cell susceptibility to bacterial infection and inflammation.

The airway epithelium represents the first line of host defense against environmental pathogens and antigens. Non specific and specific mechanisms are involved in clearing those foreign substances from the lung, in particular mucocilliary clearance, inflammatory cytokines (e.g., IL-8) and anti microbial peptides (e.g., β defensin-2) production. We showed that knocking down FABP5 resulted in increased susceptibility to bacterial infection with decreased β defensin-2 production and increased IL-8 production whereas FABP5 overexpression increased airway epithelial cell defense (e.g., increased β defensin-2 production) and reduced IL-8 production. In agreement with our results, a previous study has demonstrated that CS can modulate human gingival epithelial cells function by suppressing β defensin-2 and enhancing IL-8 production [29]. To further enhanced the significance of our findings in the context of COPD, the genomic copy number variation of β defensin-2 encoding genes has been shown to have a functional role in airway epithelial cells in response to *P. aeruginosa* infection and to be associated with COPD pathogenesis [30].

To understand the mechanism behind FABP5 immunomodulatory function, we questioned whether the peroxisome proliferator-activated receptor (PPAR)-γ was implicated. Indeed, PPAR-γ not only regulates fatty acid storage and glucose metabolism but also exerts anti-inflammatory activity [31], [32]. Furthermore, FABP5 has recently emerged as a regulator of PPAR-γ activity [33]. Our data shows that in primary NHBE cells, FABP5 down regulation resulted in reduced PPAR-γ activity, whereas FABP5 up regulation resulted in increased PPAR-γ activity. This contradicts the observation by Babaev *et al.* that macrophage FABP5 expression suppresses PPAR-γ activity, but differences may be explained by the use of different cell types [33]. Furthermore, inhibition of PPAR-γ activity by GW9662 resulted in increased IL-8 secretion and reduced β defensin-2 mRNA expression, thus confirming the immunomodulatory role of PPAR-γ in airway epithelial cells.

However, other pathways, including Toll-like receptors (TLRs) and Nod-like receptors (NLRs), may be involved as well. Indeed, TLRs play a fundamental role in pathogen recognition and activation of innate immunity. They recognize pathogen-associated molecular patterns (PAMPs) that are expressed on infectious agents, and mediate the production of cytokines (e.g., IL-8) necessary for the development of effective immunity [24]. In addition to TLR activation [34], *P. aeruginosa* triggers Nod-like receptor Nod1 signaling in epithelial cells [35]. Furthermore, β defensin-2 expression is regulated by TLR signaling in intestinal epithelial cells [25] and in response to bacterial lipoprotein [36]. *In vitro* studies demonstrated Nod1-dependent regulation of β defensins production in epithelial cells stimulated with *Helicobacter pylori* [37]. Thus, one may postulate that TLRs and NLRs are involved in our system and that somehow FABP5 expression crosstalk with one or more of these pathways. To strengthen this argument, TLR2 and TLR4 agonists have been shown to up-regulate FABP5 expression in murine macrophages [38].

FABP5 has been associated with the metabolic syndrome. It has been shown to modulate macrophages and adipose tissue function and to contribute to systemic glucose metabolism [39], thus exerting a dramatic impact on atherosclerosis, type 2 diabetes and obesity [40]. Mice that are knockout for both the adipocyte and epidermal fatty acid-binding protein (FABP4 and FABP5, respectively) are protected against the metabolic syndrome and atherosclerosis [41], [42]. Increased serum levels of FABP5 have been shown to be associated with cardio-metabolic risk and the metabolic syndrome in humans as well [43], [44]. Interestingly, a recent study demonstrated that the metabolic syndrome is a risk factor for lung function decline [45]. Thus, it appears that excess of FABP5 is detrimental. However, our study shows that reduced FABP5 level in airway epithelial cells is detrimental against bacterial infection and, as a result, COPD exacerbations. Determining whether treating the metabolic syndrome would reduce the risk of having accelerated lung function decline or whether FABP5 expression should be increased in airway epithelial cells to prevent COPD exacerbations would be of major importance to find a sustainable treatment for COPD. FABP5 may have different functions in different tissues and different diseases.

Additionally, our work provides new opportunities for studying the role of FABP5 in bacterial infections under disease conditions such as COPD. However, *in vivo* models are required to validate FABP5/PPAR-γ as a novel immunomodulatory pathway in the airway epithelium. Ultimately, unraveling the function and regulation of FABP5 will allow us to identify new therapeutic targets for CS-mediated abnormal lung inflammation and airway injury in the pathogenesis of COPD.

**Figure 9:**
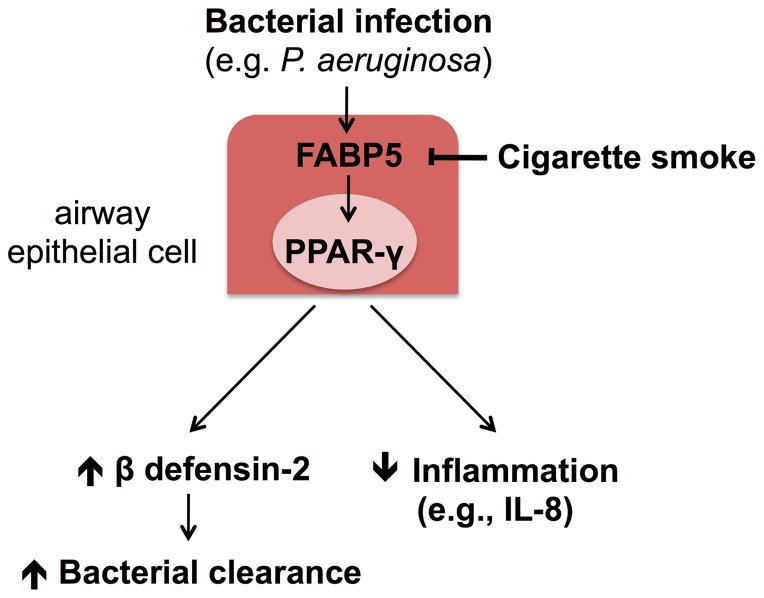
Immunomodulatory functions of FABP5 in primary NHBE cells. FABP5 exerts host defense and anti-inflammatory functions against *P. aeruginosa* bacterial infection indirectly by stimulating PPAR-γ activity. PPAR-γ activity increases β defensin-2 expression thus preventing bacterial growth and inhibits inflammatory cytokine (e.g., IL-8) production.

In summary, our study shows that CS modulates the expression of FABP5 in primary NHBE cells, thus contributing to their sensitivity to bacterial infection. Our results are in agreement with previous observations that CS alters the innate immune system, in particular the airway epithelium host defenses (e.g., β defensin-2 and IL-8). The susceptibility of smokers and COPD patients to CS-dependent diseases may be related to FABP5 expression in airway epithelial cell and its modulation of PPAR-γ activity during bacterial infection (Figure 9).

## Supporting Information

Figure S1 **Successful knock down and overexpression of FABP5 in primary NHBE cells. A.** FABP5 knock down significantly reduced FABP5 mRNA in FABP5 shRNA treated cells as compared to Firefly luciferase shRNA treated cells. **B.** FABP5 overexpression significantly increased FABP5 mRNA as compared to cells overexpressing GFP. **C.** Western blotting of FABP5 demonstrated inhibition of FABP5 protein in FABP5 shRNA treated cells and enhanced FABP5 expression in cells overexpressing FABP5. Equal amounts of protein were loaded. *n* = 3 replicates. Data are expressed as means ± SEM.
(TIFF)
