# Supplementary figures and images for: Cigarette Smoke Decreases Airway Epithelial FABP5 Expression and Promotes Pseudomonas aeruginosa Infection
Source: PLoS One. 2013 Jan 22;8(1):e51784. doi: 10.1371/journal.pone.0051784 (PMC3551956; doi:10.1371/journal.pone.0051784)

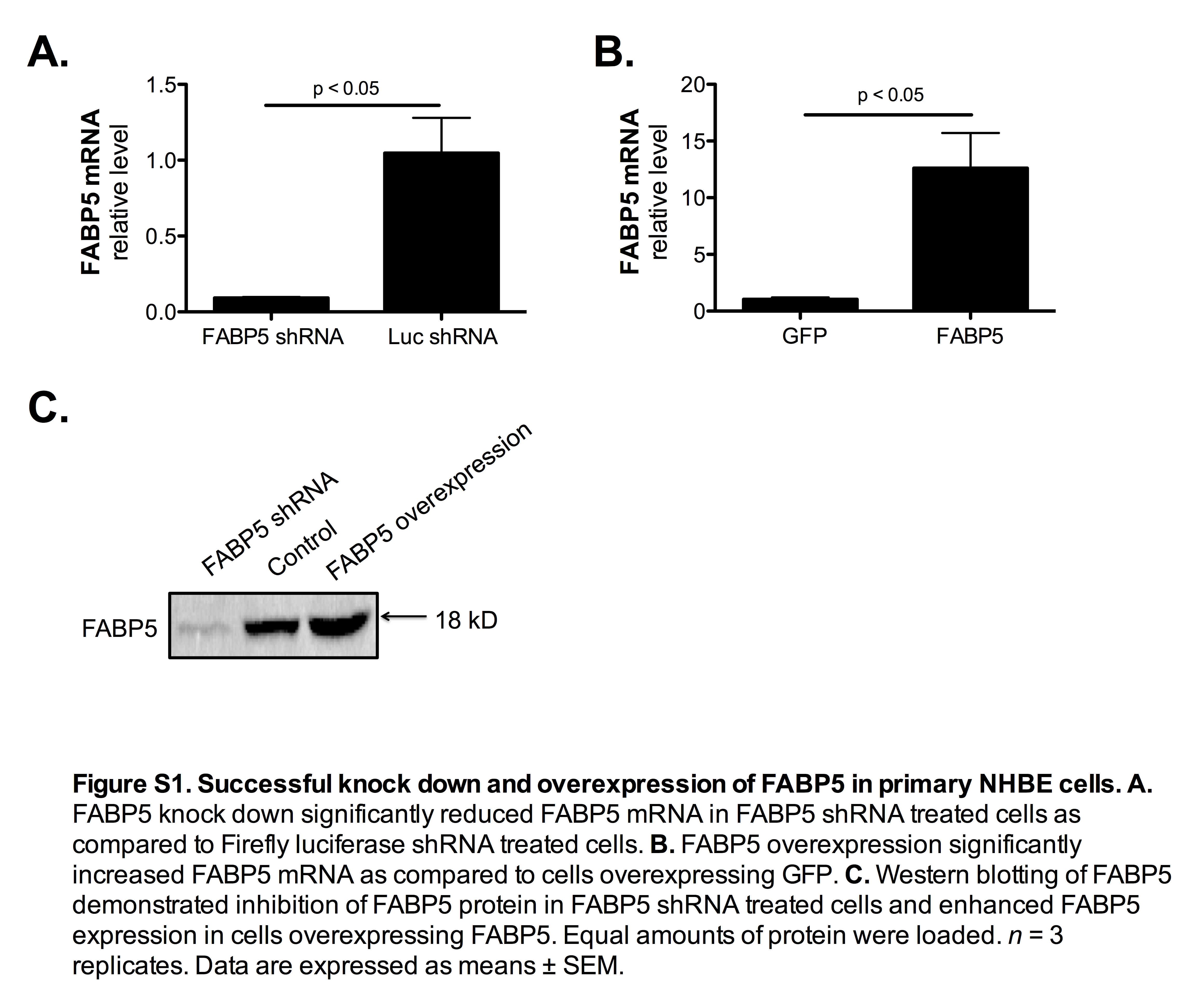

Supplement: Figure S1 — Successful knock down and overexpression of FABP5 in primary NHBE cells. A. FABP5 knock down significantly reduced FABP5 mRNA in FABP5 shRNA treated cells as compared to Firefly luciferase shRNA treated cells. B. FABP5 overexpression significantly increased FABP5 mRNA as compared to cells overexpressing GFP. C. Western blotting of FABP5 demonstrated inhibition of FABP5 protein in FABP5 shRNA treated cells and enhanced FABP5 expression in cells overexpressing FABP5. Equal amounts of protein were loaded. n = 3 replicates. Data are expressed as means ± SEM. (TIFF) [file pone.0051784.s001.tiff]
